# Draft Genome Sequence of a Polyhydroxyalkanoate-Producing Bacillus cereus Strain Isolated from Nuevo Leon State, Mexico

**DOI:** 10.1128/mra.00269-22

**Published:** 2022-06-02

**Authors:** Maria Elizabeth Alemán-Huerta, Raul E. Martínez-Herrera, Temidayo Oluyomi Elufisan, Fátima Lizeth Gandarilla-Pacheco, Isela Quintero-Zapata, Miguel Ángel Reyez-López, Erick de Jesús de Luna-Santillana

**Affiliations:** a Universidad Autónoma de Nuevo León, Facultad de Ciencias Biológicas, Instituto de Biotecnología, San Nicolás de los Garza, Nuevo León, México; b Tecnológico de Monterrey, Escuela de Ingeniería y Ciencias, Monterrey, Nuevo León, México; c Instituto Politécnico Nacional, Centro de Biotecnología Genómica, Reynosa, Tamaulipas, México; d Universidad Nacional Autonoma de Mexico, Centro de Ciencia Genomica, Cuernavaca, Morelos, México; University of Southern California

## Abstract

Polyhydroxyalkanoates (PHA) are microbially produced biopolymers which are biodegradable and biocompatible. These compounds produced by microorganisms have been described as a potent alternative to synthetic plastics, which are often recalcitrant. Here, we report the draft genome sequence of a PHA-producing Bacillus cereus isolated in our laboratory.

## ANNOUNCEMENT

*Bacillus* species are known to produce a wide variety of beneficial metabolites, among which are polyhydroxyalkanoates (PHA). We isolated a novel strain of Bacillus cereus from garden soil in San Nicolas de los Garza, Nuevo Leon, Mexico (25°43′38″N, 100°18′37″W), via serial dilution to a factor of 8. This dilution was plated onto a nutrient agar plate. The colonies which appeared on this plate were used for genomic DNA extraction for the identification of the colony. We observed that these colonies yielded a large amount of PHA under stress conditions (1, 2). Thus, we decided to sequence and analyze the genetic basis on which it produces biopolymers.

B. cereus 4N was grown to the logarithmic phase in lysogeny broth at 28°C; a portion of this culture was used for the extraction of genomic DNA. Genomic DNA was extracted using the Wizard genomic DNA extraction kit (Promega, USA) according to the manufacturer’s instructions. To identify the bacterium, we amplified the 16S rRNA region of the Bacillus cereus genome using the primer set 8F (5′-AGAGTTTGATCCTGGCTCAG-3′) and 1492R (5′-ACGGCTACCTTGTTACGACTT-3′) as described by Mizuno et al. (3). The identity of B. cereus 4N was confirmed by a BLAST search analysis on the NCBI website, which yielded an identity of 99.88% with Bacillus cereus strain LL-1.

The genomic DNA was sequenced at the Laboratorio Nacional de Nutrigenómica y Microbiómica Digestiva Animal-IPN or National Laboratory of Nutrigenomics and Animal Digestive Microbiomics-IPN using 500 ng of the submitted genomic DNA. The DNA was quantified using the Qubit double-stranded DNA (dsDNA) high-sensitivity (HS) assay kit on the Qubit fluorometer (Thermo Fisher Scientific, MA, USA). The library preparation was conducted with the Nextera Flex library kit and individual indices for bar coding, using the Illumina reference (10000000254) as a guide. The library was sequenced using Illumina MiniSeq technology.

A total of 12,710,962 paired-end 145-bp reads were obtained, with a sequencing depth of >300X, after a quality check using FastQC v0.11.9 (4). Default parameters were used for all software unless otherwise stated. The raw reads were trimmed using Trimmomatic v0.32 to remove adaptors and improve the sequence quality (5). The trimmed reads were assembled using SPAdes v3.15.3 on the KBase online platform (6, 7). The obtained contigs from the assembled reads were reduced to 27 contigs using the Medusa combo online genome scaffolder (8) with a single-contig Bacillus cereus isolate as a reference. The reduced contigs were annotated using Prokka v1.14.5 (9), and the publicly available genome was annotated using the NCBI Prokaryotic Genome Annotation Pipeline (PGAP) (10). The genome comprised 6,213,840 bp in 27 contigs with an *N*_50_ value of 5,994,343 bp and a GC content of 34.69%. The genome contains 6,084 genes and 6,388 coding sequences (CDS), with 267 amino acids, 6,084 protein-coding sequences, and 67 RNAs (7 5S, 1 16S, and 1 23S rRNAs). B. cereus 4N has 53 tRNAs and 1 CRISPR array.

**FIG 1:**
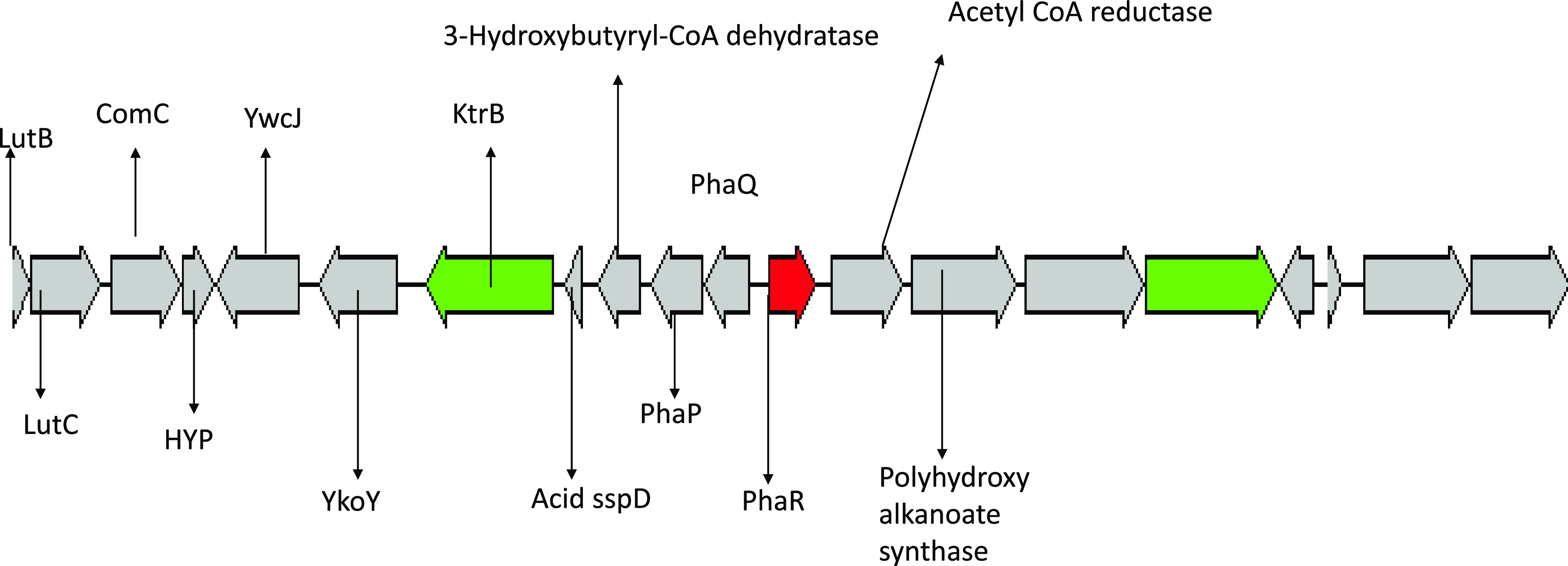
Image for the operon in Bacillus cereus 4N for the gene associated with the production of polyhydroxyalkanoate.

Five genes that are essential for the metabolism of PHA were found in an operon using the RAST annotation tool, as shown in Fig. 1. These genes include *PhaP*, *PhaR*, *PhaQ*, and polyhydroxyalkanoate synthase and 3-hydroxyl butyrate-coenzyme A (CoA) hydratase genes.

### Data availability.

The draft genome sequence of B. cereus 4N was deposited at GenBank under accession number JALBCM010000000; the project data are available under BioProject accession number PRJNA815817 and BioSample accession number SAMN26643023. The raw draft genome data were deposited in the Sequence Read Archive (SRA) under SRA accession number SRR18331562. The 16S rRNA sequence has been deposited at the NCBI website under accession number MH404097.1.
